# Polyphenols and Novel Insights Into Post-kidney Transplant Complications and Cardiovascular Disease: A Narrative Review

**DOI:** 10.3389/fcvm.2021.751036

**Published:** 2021-11-16

**Authors:** Nicolas I. Bustos, Camilo G. Sotomayor, Robert A. Pol, Gerjan J. Navis, Stephan J. L. Bakker

**Affiliations:** ^1^Faculty of Medicine, Institute of Biomedical Sciences, University of Chile, Santiago, Chile; ^2^Division of Nephrology, Department of Internal Medicine, University Medical Center Groningen, University of Groningen, Groningen, Netherlands; ^3^Radiology Department, Clinical Hospital University of Chile, University of Chile, Santiago, Chile; ^4^Division of Transplantation Surgery, University Medical Center Groningen, University of Groningen, Groningen, Netherlands

**Keywords:** polyphenols, resveratrol, kidney transplantation, delayed graft function, reperfusion injury, calcineurin inhibitors, Mediterranean diet

## Abstract

Kidney transplantation is the preferred treatment for end-stage kidney disease. It is, however, not devoid of complications. Delayed graft function related to ischemia-reperfusion injury (IRI), calcineurin inhibitor (CNI) nephrotoxicity, diabetes, and a particularly high-rate cardiovascular disease (CVD) risk, represent important complications following kidney transplantation. Oxidative stress and chronic low-grade inflammation are mechanisms of disease incompletely abrogated in stable kidney transplant recipient (KTR), contributing to the occurrence of these complications. Polyphenols, bioactive compounds with recognized antioxidant and anti-inflammatory properties have been strongly associated with prevention of CVD in the general population and have been shown to decrease IRI and antagonize CNI nephrotoxicity in animal experimental models, therefore they may have a role in prevention of complications in KTR. This narrative review aims to summarize and discuss current evidence on different polyphenols for prevention of complications, particularly prevention of CVD in KTR, pointing toward the need of further studies with potential clinical impact.

## Introduction

Polyphenols are one of the numerous groups of metabolites distributed in the plant kingdom, mainly in fruits, vegetables, and red wine (1). Chemically, polyphenols are defined as aromatic compounds with phenolic structural features. There are more than 8,000 phenolic structures described, which can be divided into several sub families, such as flavones, flavonols, isoflavones, and phenolic acids, according to their biochemical properties, source of origin, and biological function (2). Polyphenols, are bioactive compounds with a strong direct antioxidant effect by scavenging reactive oxygen species (ROS) and reactive nitrogen species (RNS), thus counterbalancing oxidative stress (3).

Kidney transplantation is the preferred treatment for end-stage kidney failure, however despite all advances in the field, kidney transplant recipients (KTR) still have higher morbidity and mortality rate compared to the general population (4). Although kidney transplantation aims to restore kidney function, it should be noted that it incompletely mitigates ongoing mechanisms of disease, including inflammation, oxidative stress, and impaired metabolic homeostasis (5). In outpatient, otherwise stable KTR, an aggregate of factors inherent to the post-kidney transplant milieu such as chronic low-grade immunologic response to the kidney graft, long-term nephrotoxicity associated to the use of calcineurin inhibitors (CNI), and elevation of serum uric acid (SUA), resulting in renal function impairment, contribute to perpetuate redox imbalance, and chronic low-grade inflammation (5, 6).

Oxidative stress and inflammation have been proposed to play an important role in the underlying pathophysiology of post-kidney transplant complications, including delayed graft function, immunosuppressive drugs-associated toxicity, diabetes, and cardiovascular diseases (CVD) (7), which is particularly important in this setting as it represents the leading cause of death in KTR with a functioning graft (8, 9).

Research in the last decades strongly support a role of polyphenols in the prevention of CVD in the general population (10). Such role has been attributed, in part to the anti-oxidant capacity of polyphenols, but also to alternative properties, including modulation of cell signaling pathways, antithrombotic and anti-inflammatory effects, vasodilatory properties, protection against LDL-oxidation, and prevention of endothelial dysfunction (10–12).

Therefore, it is plausible to propose that polyphenols might have a beneficial role in KTR by preventing complications in which oxidative stress and inflammation are key mechanisms of disease. The aim of this narrative review is to explore the existing evidence on the role of polyphenols in post-kidney transplantation in terms of prevention of short and long-term complications, including delayed graft function, CNI associated nephrotoxicity, and CVD. Findings may have a clinical impact since polyphenols could represent a cost-effective therapeutic option with scarce adverse effects. To our knowledge, no previous reviews discussing the potential role of polyphenols in KTR setting have been published.

## Polyphenols and Cardiovascular Protective Effects: Molecular Mechanisms

**Figure 1:**
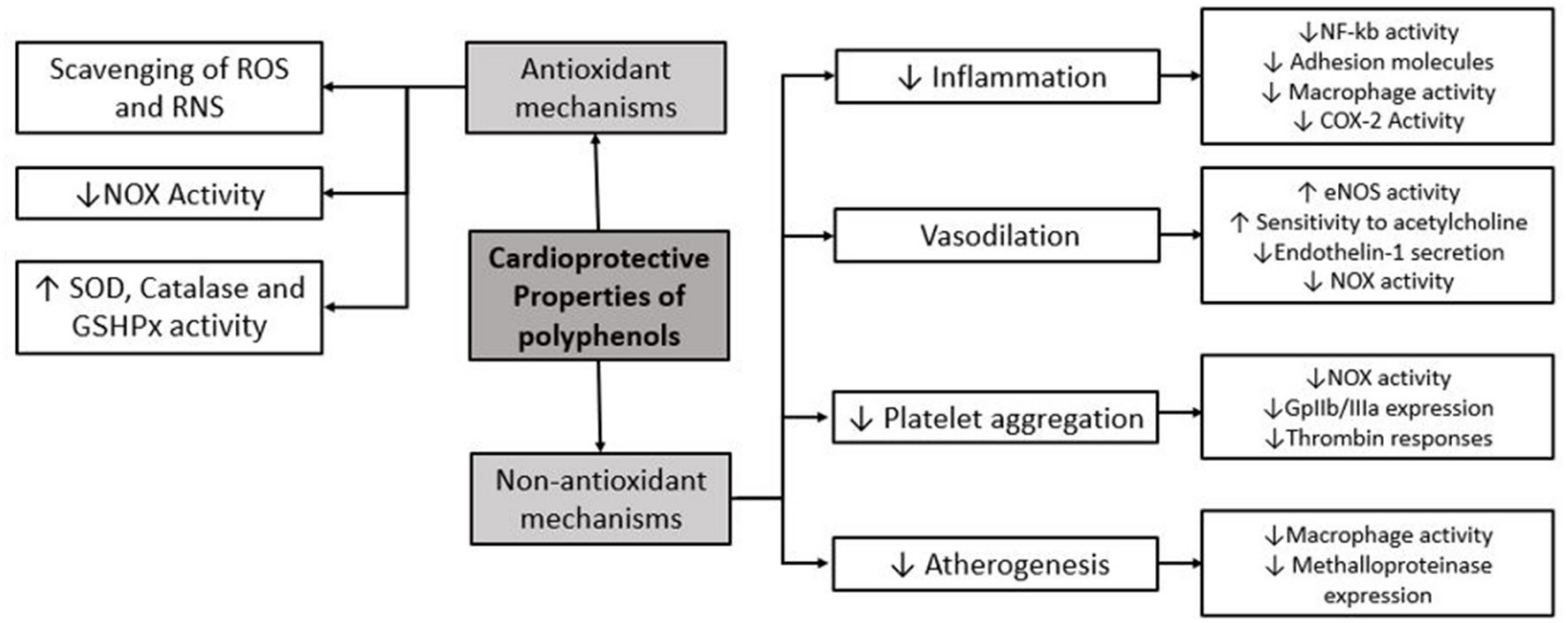
Main molecular mechanisms of protective effects of polyphenols. ROS, reactive oxygen species; RNS, reactive nitrogen species; NOX, NADPH oxidase; SOD, superoxide dismutase; GSHPx, glutathione peroxidase; COX-2, cyclooxygenase-2.

Protective effects of polyphenols are explained by antioxidant as well as alternative mechanisms (Figure 1). The antioxidant properties of polyphenols include scavenging ROS and RNS, based on the donation of a hydrogen from free radicals generating phenoxyl radicals that are stabilized by polyphenolic molecular structure. Moreover, polyphenols directly inhibit some enzymes involved in ROS generation such as NADPH oxidase (NOX) and upregulate endogenous antioxidant enzymes like superoxide dismutase (SOD) and catalase (10). Polyphenols also have non-antioxidant properties, which are involved in cardiovascular protection. These include (1) vasodilatory effect by enhancing endothelial NO release and endothelial sensibilization to acetylcholine and inhibiting vasoconstrictor enzymes such NOX (2, 10) anti-inflammatory effect by inhibition of NF-kB through different pathways including modulation of redox status, inhibition of kinases and other proinflammatory molecules, and also by decreasing proinflammatory gene expression involving transcriptional modulation (3, 13) anti-aggregation properties; and (4) antiatherogenic effect, which also confers a cardioprotective role (10).

## Renal Ischemia and Reperfusion Injury: Pathophysiology, Clinical Complications, and Treatment

Renal ischemia-reperfusion injury (IRI) is a complex and inevitable phenomenon in kidney transplantation that strongly impacts in post-transplant complications, contributing to poor long term graft outcomes (14). Ischemia-reperfusion injury involves different mechanisms including activation of cell death programs, transcriptional reprogramming, endothelial dysfunction, and activation of the innate and adaptive immune system (15).

At a cellular level two phases are distinguished. First, there is damage occurring during ischemia, in which oxygen delivery decreases and cell switches to anaerobic metabolism, with ATP drop and intracellular acidosis. In this phase lysosomal membrane is destabilized, with enzymes leakage, Na/K bomb decreases its activity leading to accumulation of Na and water, and finally inducing cell edema. Also, calcium efflux decreases and intracellular calcium overload activates proteases such as calpains, which are inhibited by acidosis during this phase (14, 15). In the second phase, during reperfusion oxygen rapidly increases normalizing intracellular pH and activating calcium induced calpains with proteolytic effect that impairs cell structure. Mitochondria is key during this phase, as it increases ROS production including superoxide anion from succinate accumulated during the ischemia phase. Oxidative stress contribute to structural cell damage as well as transcriptional effect leading to the activation of different cell dead programs (15).

Underlying mechanisms include crosstalk between oxidative stress and inflammation, as higher ROS production is associated to protein oxidation which triggers inflammatory molecules and different inflammatory cell pathways. At the same time tissue injury induced by ROS lead to an inflammatory process (16).

At a vascular level renal IRI involves endothelial inflammation and dysfunction, impairing microvascular permeability, promoting cell infiltration, and leukocyte activation. Also, the increased production of vasoconstrictor factor in endothelium and the reduction of NO generation during reperfusion phase lead to vasoconstriction that may impair organ reflow (14, 17).

Rapidly after IRI is initiated, innate immune system is activated, DAMPS are recognized by TLR, which activate kinases that amplify signal to transcriptional factors that induce inflammation and complement system activation. On the longer term, the adaptative immune response is activated by mechanisms that are enhanced by the inflammatory environment, finally favoring renal graft rejection and chronic fibrosis (14, 15).

Clinically IRI is associated with delayed graft function in the short-term, acute- and chronic-rejection, and chronic graft dysfunction due to interstitial fibrosis and tubular atrophy (14).

Therefore, prevention and treatment of renal IRI is critical for improving outcomes in KTR. With respect to prophylaxis, optimal donor management is essential to reduce the risk, in this phase ischemic preconditioning have been effective in reducing IRI in animal models however it have not been translated to clinical trials (18). During graft storage, hypothermic machine perfusion significantly improves outcomes (19). Many molecules have been studied accounting prevention of renal IRI including erythropoietin, Heme oxygenase 1 (HO-1), HIF-1 in experimental studies (15). Complement and innate immune system have also been tested as a therapeutic target using monoclonal antibodies that inhibits its activation during IRI, with promising therapeutic options for the future (20). Immunosuppressive drugs used in the chronic treatment in KTR counterbalance inflammation and prevent allograft rejection in which IRI is in part involved. However, direct effect of these drugs on IRI is controversial. In studies on animal models rapamycin have been described to protect from IRI in early stages through antiapoptotic effects in part by promoting kidney recruitment of natural killer T cells and by inhibition of HIF-1 (21). However, rapamycin and calcineurin have also been described to worsen the acute damage induced by IRI in animal models by direct nephrotoxic effects (22).

Anti-inflammatory and antioxidant strategies therapies with L-arginine and N-acetylcysteine have also been tested. Superoxide dismutase administrated intravenously during transplantation reduces acute rejections and improves long term outcomes (14). In this context protective properties of polyphenols may positively impact on IRI prevention at different levels, wherefore polyphenols can be proposed as a novel therapeutic tool.

## Polyphenols and Short-Term Complications: Role of Polyphenols in Prevention of Kidney Ischemia-Reperfusion Injury

Most data concerning the role of polyphenols in kidney transplantation come from *in vitro* and *in vivo* animal studies aimed to test a potential role of polyphenols on inhibiting IRI, preventing short-term complications after inducing renal ischemia by clamping the artery, followed by resumption of blood flow to simulate warm ischemia, or during hypothermia storage to simulate cold ischemia. Most relevant studies are summarized in Table 1.

**Table 1:** Overview of *in vitro* and *in vivo* studies on the effect of different polyphenols in prevention of kidney ischemia-reperfusion injury and kidney transplantation short and long term complications.

**Study design**	**Model**	**Compound**	**Result**	**References**
*In vitro* experiment of cold ischemia injury (cold storage 20 h at 4°C and rewarming). Addition of polyphenols directly to preservation solutions. No follow-up.	LLC-PK1 cultured cells	Bioflavonoids	Polyphenol meliorated cold storage—induced injury of renal tubular cells.	Ahlenstiel (23)
*In vitro* experiment of cold ischemia injury (cold storage 16 h at 4°) in kidney cell culture. Addition of polyphenols directly to preservation solutions. No follow-up.	LLC-PK1 cultured cells	Quercitin, resveratrol, BHA, and EGCG	Polyphenol prevented hypothermia-induced cell injury reducing oxidative stress.	Karhumaki (24)
*In vivo* experiment of IR (warm ischemia for 30 min). Polyphenol was administered i.v. 12 h before. Creatinine and urea was measured 4–24 h post reperfusion and renal tissue was examined.	C57/B6 mice	Curcumin (4 mg/kg i.v. by liposomal incorporation)	Reduced intracellular superoxide generation, cellular apoptosis, tissue inflammation, reduced histological injury, and improved renal function.	Rogers et al. (25)
*In vivo* experiment of IR (warm ischemia for 60 min). Polyphenol was administered 30 min before renal clamping.	Male Sprague-Dawley rats	Resveratrol (0.23 μg/kg i.g.)	Decreased oxidative stress and cell apoptosis, inhibited inflammatory response, and improved renal function.	Li et al. (26)
*In vitro* experiment of IR (cold hypoxia 24 h at 4°C and rewarming) and *in vivo* experiment with animal models. Addition of single dose of polyphenol to graft preservation solution. 1-month follow-up.	*In vitro* experiment with human kidney endothelial cells *In vivo* experiment preclinical pig model of kidney autotransplantation	Vectisol ® (2.2 mg trans-resveratrol and 1,577.8 mg cyclodextrins)	Slowed the onset of histopathological lesions, improved glomerular filtration and proximal tubular function early recovery; normalized serum creatinine and proteinuria after 1 month follow-up.	Soussi et al. (27)
*In vitro* experiment of IR (cold hypoxia 24 h at 4°C and rewarming), and *in vivo* animal autotransplantation model 3-month follow-up.	*In vitro* experiment with LLC-PK1 cultured cells. *In vivo* experiment with large white male pigs	Cyclodextrin-complexed curcumin (12 mg/ml of curcumin) added to preservation solution	CDC decreased mitochondrial loss of function, improved viability and lowered endothelial activation. *In vivo*, CDC lowered histological injury, limiting fibrosis, improved function recovery, and doubled animal survival.	Thuilier et al. (28)
*In vitro* model of endothelial cells subjected to hypoxia-reoxygenation and *in vivo* animal model of warm ischemia and allotransplantation (cold storage 6 h at 4°) Intraperitoneal injection of polyphenol 30 min before clamping or allotransplantation surgery. 1 week follow-up.	Rat model	Tannic acid (50 mg/kg i.p.)	Polyphenol improved kidney recovery after warm ischemia but not after allotransplantation (cold ischemia). It also limited cytotoxicity and ROS production in renal biopsies, and it promoted endothelial cell migration and proliferation during hypoxia.	Alechinsky et al. (29)
*In vitro* experiment of demonstration of autophagy and associated fibrosis. *In vivo* experiment with kidney allotransplantation animal model cells were cultured 16 weeks after transplantation.	*In vitro* experiment with HUVECs of patients with chronic allograft dysfunction and *in vivo* experiment with rat kidney transplantation model	Curcumin (200 mg/kg i.g.)	Curcumin inhibited IL-6-dependent Endothelial to mesenchymal transition by inducing autophagy *in vitro* and *in vivo*. Alleviated allograft fibrosis and deterioration of renal functional, and prolonged survival.	Zhou et al. (30)

*BHA, butylated hydroxyanisol; CDC, cyclodextrin-complexed curcumin; EGCG, Epigallocatechin gallate; HUVECs, human umbilical vein endothelial cells; IR, ischemia and reperfusion; LLC PK1, porcine proximal tubular epithelial cells*.

*In vitro* studies in renal cells suffering cold ischemia injury supports that addition of polyphenols to preservation cell solutions reduce oxidative stress and prevent cold ischemia induced injury (23, 24). Interesting results of *in vivo* experiment of animal kidney IRI models suggest that administration of polyphenols such as resveratrol, curcumin, and quercetin, before applying ischemia, resulted in significant reduction of short-term renal damage. This effect was observed in terms of lower oxidative stress and inflammation biomarkers levels, reduced histological injury, and prevention of renal dysfunction (25, 26, 31).

Regarding kidney transplantation setting, to our knowledge no clinical studies have been performed and only few preclinical studies in animal models have investigated the role of polyphenols in the prevention of short-term complications. Two research groups investigated the effect of polyphenol addition to organ solution preservation (cold ischemia) in a preclinical pig model of autotransplantation. Soussi et al. found that a single dose of resveratrol (in a formulation with cyclodextrin) in the static cold preservation solution had beneficial effects in terms of histological structure and graft function at 1 month post transplantation and preventing delayed graft function after 24 h of cold ischemic time (27). Thuillier et al. combined warm ischemia with static cold storage in order to mimic the level of damage found in donation after circulatory death organs, and found that addition of curcumin (in a formulation with cyclodextrin) to the preservation solution, lowered histological injury, significantly reduced fibrosis, improved renal function recovery, and doubled animal survival after 3 months of follow-up (28). Possible mechanisms proposed by authors include the strong antioxidant activity of curcumin and capacity to block inflammatory cell pathways that blunt IRI and then renal fibrosis, *in vitro* analysis of the experiment reports that cells preserved with curcumin showed less necrosis and higher mitochondrial activity suggesting that curcumin inhibit deleterious pathways induced by cold hypoxia, after reperfusion cells also presented swifter recovery of mitochondrial activity.

A more recent study evaluated in a rat model the effect of intraperitoneal tannic acid administration before renal artery clamping (warm ischemia) or after allotransplantation surgery (cold ischemia) and found that tannic acid significantly improved kidney function after warm ischemia, but not after allotransplantation. This observations can potentially be explained by the intensity of injury induced by allotransplantation, which perceivably is much higher than the injury induced by warm ischemia alone (29). As a potential mechanism for protective findings, molecular dynamic simulations showed that tannic acid efficiently interacts with biological membranes allowing inhibition of lipoperoxidation, promoting endothelial cell migration, and promoted cell regeneration during hypoxia.

Concerning chronic allograft rejection and polyphenols there is scarce evidence, a recent study investigated the effect of curcumin in rat kidney transplantation model, and reported that after 16 weeks treatment with curcumin, it alleviated allograft fibrosis and deterioration of renal function, and prolonged survival (Table 1) (30). It is described that part of the effect of curcumin is due to inhibition of IL-6-dependent endothelial to mesenchymal transition by inducing autophagy *in vivo* and *in vitro*.

To summarize, in terms of preventing kidney IRI induced by warm and cold ischemia, polyphenols have shown beneficial short-term effects concerning inflammation, histological damage, kidney function, and recipient survival. Therefore, it seems plausible that addition of polyphenols such as resveratrol or curcumin to organ preservation solutions may be beneficial in human KTR to prevent kidney IRI and complications such as delayed graft function, acute, and chronic graft rejection. To this aim it is necessary to perform clinical studies that consider a longer follow-up. Further studies should also consider particularities of polyphenols in KTR and conditions before transplantation including the plasma levels of polyphenols prior to transplantation and the effect of hemodialysis.

## Polyphenols and Immunosupressive Drugs in KTR: Role in Prevention of Calcineurin Inhibitor-Associated Nephrotoxicity

Immunosuppressive drug therapy is a cornerstone in management of KTR, reducing rejection rates, and improving graft survival. However, treatment with CNIs, which is the cornerstone of most common concurrent immunosuppressive regimens, has been associated to long-term nephrotoxicity (32) and metabolic complication such as abnormal lipid profiles and post transplantation diabetes mellitus (PTDM), which increase cardiovascular risk (33). Calcineurin inhibitor-associated nephrotoxicity is manifested by interstitial fibrosis as well as hemodynamic changes, including imbalance of vasoconstrictor mediators leading to impairment in renal function and hypertension (34). Its mechanisms are not completely understood, but oxidative stress has been reported as an important mediator in several studies (35), particularly in the stable KTR setting (36, 37).

In this context polyphenols may have a therapeutic role by reducing oxidative stress and inflammation and thus reducing nephrotoxicity and preventing its complications.

In regard to the immunomodulatory effect of polyphenols, each type of compound binds to different receptors on immune cells, triggering multiple pathways that modulate host immune system. It has been described to promote regulatory T cells differentiation, involved in immune tolerance and autoimmune control; to repress macrophages and affect activity of Th1, Th2, and Th17 cells, as well as other mechanisms. However, how this mechanisms interact in the different clinical conditions is controversial and not completely understood (13).

No studies have been performed in a kidney transplantation model and most of the studies in this area have been performed in kidney animal models of nephrotoxicity or *in vitro* experiments with renal cells, however, have not studied long term complications such as metabolic impairment as an outcome. Studies accounting for the effect of addition of polyphenols to cyclosporin and tacrolimus regimens are summarized in Table 2.

**Table 2:** Overview of the studies on the effect of polyphenol in prevention of calcineurin inhibitors induced nephrotoxicity.

**Study design**	**Subjects**	**Compounds**	**Result**	**References**
*In vivo* experiment. Oral administration of CNI for 10 days followed by oral administration of polyphenol for 21 days.	Wistar rat model of cyclosporine-induced kidney injury (*n* = 50)	Cyclosporine A (50 mg/kg) and *Zingiber officinale* (Ginger) polyphenols (100, 200, and 400 mg/kg p.o.)	Restored normal homeostasis of the antioxidant system measured using GSH and SOD, prevented kidney weight loss and normalized serum electrolytes, creatinine, and urea.	Adekunle et al. (38)
*In vitro* and *in vivo* experiment. Daily oral administration for 2–4 weeks.	Human CD4 T cells isolated from PBMC and HRPTEpiCs for *in vitro* study and murine skin transplant model for *in vivo* study (*n* = 18)	Tacrolimus (2 mg/kg) and resveratrol (100 mg/kg p.o.)	Resveratrol provides additional immunosuppression effect to tacrolimus treatment by suppressing Th17 cells. Combination also prolonged survival duration of skin allograft.	Doh et al. (39)
*In vivo* animal experiment. Daily oral administration of CNI + polyphenol for 28 days.	Rat model of cyclosporine-induced chronic nephrotoxicity (*n* = 28)	Cyclosporine A and tea polyphenols	Decreased cell apoptosis, ameliorated fibrosis, and renal dysfunction induced my cyclosporine.	Shi et al. (40)
*In vivo* animal experiment. CNI + polyphenol i.g. administration for 28 days.	Rat model of cyclosporine A-induced chronic nephrotoxicity (*n* = 40)	Cyclosporine A (15 md/kg/d s.c) and tea polyphenols (80 mg/kg/d i.g.)	Tea polyphenols ameliorated fibrosis and renal dysfunction induced by cyclosporine, in part by inhibiting TGF-beta 1 expression.	Shi et al. (41)
*In vivo* animal experiment. CNI + polyphenols oral administration for 21 days.	Rat model of cyclosporine A-induced chronic nephrotoxicity (*n* = 40)	Cyclosporine A (15 mg/kg/d s.c) and provinol (40 mg/kg/d p.o)	Provinol addition improved renal function by decreasing ROS, iNOS, and NF-kB expression.	Buffoli et al. (42)
*In vivo* animal experiment. Oral administration of polyphenol for 3 days followed by CNI por 5–21 days.	Rat model of cyclosporine A and tacrolimus induced nephrotoxicity (*n* = 40)	Cyclosporine A (25 mg/kg/d) or tacrolimus (2 mg/kg/d) and polyphenolic extract of *Camellia sinensis* 0.1% (green tea)	Polyphenols prevented structural impairment and renal dysfunction induced by tacrolimus or cyclosporine by decreasing oxidative stress and lipid peroxidation.	Zhong et al. (43)
*In vivo* animal experiment. CNI + polyphenol i.p administration for 7 days.	Rat model of cyclosporine A induced nephrotoxicity (*n* = 31)	Cyclosporine A (25 mg/kg /day, s.c) and resveratrol (10 mg/kg per day, i.p.)	Resveratrol prevented tubular damage and leukocyte infiltration and protected against the endothelial/vascular dysfunction induced by cyclosporine.	Bekpinar et al. (44)
*In vitro* and *in vivo* animal experiment. CNI + polyphenol administration for 14 days.	HK-2 human proximal tubule epithelial cell line for *in vitro* experiment and mice model of ciclosporin A induced nephrotoxicity (*n* = 72)	Ciclosporin A (15 mg/kg/day sc) and curcumin (15/mg/kg/day)	Curcumin attenuated cyclosporine induced renal fibrosis by enhancing Klotho expression and inhibiting TGF-b signaling.	Hu et al. (45)
*In vivo* animal experiment. Polyphenol administration 3 days followed by CNI administration for 21 days.	Rat model of cyclosporine A and tacrolimus induced nephrotoxicity (*n* = 16)	Cyclosporin A (25 mg/kg/d i.g.) and polyphenolic extract of *Camellia sinensis* 0.1% (green tea)	Green tea polyphenols attenuated CSA induced renal injury, stimulated mitochondrial biogenesis, and improved renal function.	Rehman et al. (46)
*In vivo* animal experiment. CNI and polyphenol oral administration 1 day before and for 21 days concurrently with CNI.	Rat model of cyclosporine A induced nephrotoxicity (*n* = 48)	Cyclosporine A (20 mg/kg/day s.c for 21 days) and resveratrol (2–5–10 mg/kg/day p.o.)	Resveratrol significantly improved tissue and urine total nitric oxide levels, reduced renal oxidative stress, prevented the alterations in renal morphology, and improved renal function.	Chander et al. (47)
*In vivo* animal experiment. CNI + polyphenol oral administration for 21 days.	Rat model of cyclosporine A induced nephrotoxicity (*n* = 80)	Cyclosporin (15 mg/kg/day, s.c.) and provinol flavonoids (40 mg/kg/day p.o.)	Provinol prevented nephrotoxicity, morphological and biochemical ciclosporin-induced alteration by antiapoptotic effects.	Rezzani et al. (48)

*CNI, calcineurin inhibitor; GSH, reduced glutathione; HK2, human kidney 2 cells; HRPTEpiCs, human renal proximal tubular epithelial cells; iNOS, induced isoform of nitric oxide synthase; PBMC, peripheral blood mononuclear cells; ROS, reactive oxygen species; SOD, superoxide dismutase*.

These studies concluded that cyclosporine and tacrolimus—induced nephrotoxicity can be significantly prevented by adding polyphenols, at least in animal models *in vitro* and *in vivo*. Experiments showed a significant decrease in oxidative stress and inflammation markers, prevention of endothelial dysfunction, reduction in cell fibrosis, and improvement of renal function when polyphenols are co-administered to cyclosporine and tacrolimus. However, follow-up of these studies was no longer than 1 month. Thus, whether this protective effect remains after chronic drug use is unknown.

Only one study included polyphenols and cyclosporine effect in transplantation setting, using an *in vivo* murine skin transplant model, and demonstrated that addition of resveratrol to tacrolimus treatment prolonged survival of the skin allograft (39). With *in vitro* and *in vivo* experiments, the researchers also found that resveratrol provides additional immunosuppression effect by suppressing Th17 cells response. This is particularly interesting because tacrolimus, despite being an important immunosuppressant of T cell responses, is inadequate to suppress Th17 cells (49) which has been associated to increased allograft rejection. This is in agreement with more recent studies that support the role of resveratrol in the attenuation of stimulated T-cells by inhibit the expression and activation of mTOR mediated cellular signaling (50). Therefore, addition of resveratrol to a tacrolimus regimen may represent an adjunctive therapy option for patients undergoing organ transplantation.

To conclude, polyphenols may have therapeutic role as co-adjuvant in CNI immunosuppressive treatment after transplantation, by reducing nephrotoxicity, oxidative stress, and by potentiating the immunosuppressive response. More studies should be performed in animal or human models of solid organ transplantation particularly kidney transplantation, to support this hypothesis and to account for the potential role of polyphenol in reducing the cardiovascular risk induced by therapy.

## Dietary Polyphenols in KTR: Mediterranean Diet, An Approach to the Role of Polyphenol Into Prevention of Cardiovascular Complications

The demonstration of the properties of polyphenols *in vivo* has encouraged an extensive study of dietary sources of polyphenols within the cardiovascular research field. Dietary polyphenols, have shown a role in the regulation of inflammation (13) and have proven immunomodulatory and anti-inflammatory effects, lowering the risk of CVD. The main sources of polyphenols in diet are fruits such specially berries, vegetables, seeds, grains, nuts, coffee, and red wine (51).

The Mediterranean diet (MD) is a dietary pattern traditionally consumed by inhabitants of Mediterranean regions and is characterized by a high intake of fish, fruit, vegetables, legumes, nuts, and olive oil, and a low intake of meat products. To date, the MD is one of the most extensively studied dietary styles, and has demonstrated to reduce the risk of CVD, diabetes, and mortality in the general population (52, 53). Mediterranean diet has also shown protection against lipid peroxidation and inflammation, and it is the most recommended diet for KTR (54). On the other hand, the western diet, well-recognized for its proinflammatory effects related to high sugar and saturated fat intake, reduced content in fiber, complex carbohydrates, micronutrients, and lack of bioactive molecules such as omega-3 polyunsaturated fatty acids and polyphenols (55), is recommended to be avoided in KTR.

Considering the scarcity of studies on the effect of polyphenols in stable KTR, the role of polyphenols in preventing cardiovascular complications after kidney transplantation have not yet been studied in clinical nor preclinical models. Taking in consideration the lack of evidence concerning the direct effect of dietary polyphenols in stable KTR, we considered studies accounting for the effect of the polyphenols-rich MD, as it has been investigated in human kidney transplantation. Therefore, it may be considered as indirect evidence for beneficial effects of polyphenols in KTR. However, it must be considered that MD is rich in many other elements that also protect against CVD, for example fibers which are related to better glycemic control, and have intrinsic antioxidant and anti-inflammatory effect; omega 3 polyunsaturated fatty acid which is found in olive oil and fish; and micronutrients such as vitamin D, magnesium, and zinc, known for its protective effect against CVD and its anti-inflammatory properties (54). Although the protective effect of MD in KTR, cannot be attributed exclusively to polyphenols considering the existing evidence, it may hold a plea for future studies evaluating the contribution of dietary polyphenols to reduce the cardiovascular burden in KTR.

We found six prospective studies on human adult KTR that evaluated the short- and long-term effect of the MD (Table 3). The subjects were KTR with a functioning graft, representing the stable outpatient group whose main clinical problems are cardiovascular complication and chronic graft failure. The most extensive study accounted 632 patients, who were observed for a median follow-up of 5.4 years. This study found that MD was associated with graft survival and better kidney function (56).

**Table 3:** Overview of prospective clinical studies on the effect of Mediterranean diet in kidney transplant recipient.

**Study design**	**Subjects**	**Result**	**References**
Prospective observational study. Median 5.4 years follow-up.	Six hundred and thirty-two adult kidney transplant recipients with a functioning graft for ≥1 year.	Mediterranean diet score was inversely associated with graft failure, kidney function decline, and graft loss.	Gomes-Neto et al. (56)
Randomized prospective study. 6 weeks follow-up.	Thirty-seven adult kidney transplant recipients with stable graft function, non-smokers. Twenty-one patients in MD and 16 patients on low-fat diet.	MD improved plasma oxidative status (significantly Increased SOD activity, oleic acid, and decreased CAT, GSHPx, linoleic acid, and TBARS, a peroxidation marker). Total cholesterol levels and triglycerides were significantly reduced.	Stachowska et al. (57)
Prospective observational study. 1 year follow-up.	One hundred and sixty adult renal allograft recipients with no existing metabolic syndrome or diabetes mellitus.	Mediterranean dietary pattern was associated with a reduced risk of metabolic syndrome at 1 year.	Nafar et al. (58)
Prospective cohort observational study. Median 4.0 years follow-up.	Four hundred and sixty-eight adult kidney transplant recipients with functioning graft for >1 year.	Hight MD score was associated with lower risk of PTDM, lower triglycerides levels, and higher HDL concentration.	Oste et al. (59)
Randomized prospective study. 6 months follow-up.	Thirty-seven adults kidney transplant recipients with stable graft function, non-smokers. Twenty-one patients in MD (study group) and 16 patients on low fat diet (control group).	MD significantly reduced cholesterol levels in the group of young and middle-aged patients. This tendency was not observed in elderly patients nor in patients with severe baseline dyslipidemia.	Stachowska et al. (60)
Prospective observational study. 10–12 weeks follow-up.	Seventy-eight adults renal transplant recipient, normolipidemic, and hyperlipidemic with stable graft function and free of cardiovascular disease.	MD led to a significant reduction in total cholesterol levels, triglycerides, low-density lipoprotein (LDL)-cholesterol whereas HDL-cholesterol levels remained unchanged.	Barbagallo et al. (61)

*CAT, catalase; GSHPx, glutathione peroxidase; MD, Mediterranean diet; PTDM: post transplantation diabetes mellitus; SOD, superoxide dismutase; TBARS: thiobarbituric acid reactive substance*.

Adherence to MD in KTR improved the oxidative status of blood (57), reduced risk of metabolic syndrome (58) and PTDM (59), and improved the lipid profile by decreasing of total cholesterol and triglycerides levels (57, 59–61). However, after long term follow-up, the effect on lipid profile was only significant in young and middle-aged patients without severe baseline dyslipidemia, supporting a preventive rather than a therapeutic role of MD (60). In addition to MD, considering the specific effect of fruit and vegetables consumption in KTR, a large cohort study found that vegetable, but not fruit, intake was associated with a lower risk of PTDM in KTR after 5.2 years of follow-up (62).

In summary, MD seems to be the preferred diet for KTR in terms of graft survival and reducing cardiovascular risk. However, more studies are required to determine the extent of the effect of each dietary component, with emphasis on the effect of dietary polyphenols.

No studies have assessed the levels of serum polyphenols prior and after transplantation, patients before transplantation might have low dietary intake due to poor specific intake as well as dietary restriction of potassium in end stage kidney disease thus low intake of fruits and vegetables. In addition maintenance hemodialysis decreases the concentration of polyphenols in plasma (63), thus it can be hypothesized that non-pre-emptive patients at admission for transplantation have low levels of polyphenols in blood, which may in turn impact clinical outcomes, specially short-term complications. On the other side there is evidence that after transplantation dietary intake of fruits and vegetables is low (62), thus polyphenols might remain low in the stable post transplantation setting. In fact when asked specifically, KTR state to stick to a diet low in vegetables and fruits because they assume that also after transplantation a low potassium diet is better for their health (64). This is an important clinical point in the assessment of KTR that might be missed by some clinicians.

In concern to the levels of polyphenols in KTR, weather the beneficial effect of polyphenol suppletion is due to correction of some deficit or due to pharmacological effect irrespective of prior status represent a question to be answered in future studies.

## Pharmacological Consideration for Further Clinical Approach

Despite the extensive success of polyphenols in *in vitro* and preclinical studies, interventional studies have not been consistent enough to reproduce the same positive results. One relevant concern in pharmacological application is the route of administration and the necessary dose to reach an optimal biological activity, which is impaired by the limited bioavailability of polyphenols. Pharmacokinetics and its bioavailability depend on the metabolism, absorption, distribution, and excretion. In oral mucosa and the gastrointestinal tube complexes mechanism are involved in metabolism and absorption processes including cotransporters, efflux pumps, local microbiota, and enzymes, which are also modulated by polyphenols itself (10, 65). The extensive pharmacokinetics associated to polyphenol metabolism, eventual inactivation during this process and individual variability of pharmacokinetic parameters among each compound, represent a challenge for clinical studies to demonstrate effectiveness. Chemical properties for example the hydrophobic nature of some polyphenols such as resveratrol and curcumin considerably reduces its bioavailability. At present, there are several studies aimed to achieve better polyphenol bioavailability by new systems of delivery of each compound, including liposomal formulation, prodrugs, or coadministration with absorption enhancers (66, 67). In kidney transplantation models, studies that evaluated the *in vivo* effect of polyphenols addition to preservation solutions, used indeed formulation with cyclodextrin to enhance organ delivery of resveratrol and curcumin with promising results (27, 28). Cyclodextrin are cyclic oligosaccharides that, in formulation with these molecules, confers solubilization by taking the hydrophobic part of a molecule into their cavity, thus enhancing delivery to the target organ and causing an efficient cell uptake of the polyphenol (27, 28).

## Conclusion

Despite the scarcity of clinical and preclinical studies aimed to test the role of polyphenol in KTR, the existing evidence in animal models show that polyphenols prevent kidney IRI and CNI nephrotoxicity, in part by counterbalancing oxidative stress and inflammation. With respect to cardiovascular complications, no studies have been performed to date, though indirect evidence through dietary interventions using MD in KTR, points toward a role in prevention of metabolic complications that increase cardiovascular risk.

Polyphenols participate in counteracting mechanisms of disease in KTR, and it is plausible to hypothesize a role in KTR at two levels in future clinical trials:

### Pre-transplantation Setting

Polyphenols addition to preservation solution to prevent IRI in KTR, resulting in diminished delayed graft function and improved graft survival.

### Post-transplantation Setting

Polyphenols as coadjuvant therapy in KTR to prevent CNIs-induced nephrotoxicity, with complementary immunosuppressive effects, and potentially reduce cardiovascular risk and prevent CVD.

In conclusion, there is an unexplored and promising field of research. More human observational and interventional studies are warranted, accounting for the direct effect of polyphenols in management of KTR and their impact in prevention of chronic complications such as CVD. Both dietary and pharmacological approaches could theoretically be promoted considering pharmacological limitation and new methods to enhance bioavailability of polyphenols. Now, for clinic assessment of KTR is important to ensure an adequate dietary intake, and given the magnitude of the problem, pharmacological suppletion might be necessary, being essential to further identify in whom and how much.

## Author Contributions

NB, CS, and SB contributed to the conception of the paper, searched, and interpreted data. NB and CS wrote the first manuscript. SB, RP, and GN revised and adapted the manuscript and contributed to the clinical vision. SB is the guarantor of this work. All authors contributed to manuscript revision, read, and approved the submitted version.

## Conflict of Interest

The authors declare that the research was conducted in the absence of any commercial or financial relationships that could be construed as a potential conflict of interest.

## Publisher's Note

All claims expressed in this article are solely those of the authors and do not necessarily represent those of their affiliated organizations, or those of the publisher, the editors and the reviewers. Any product that may be evaluated in this article, or claim that may be made by its manufacturer, is not guaranteed or endorsed by the publisher.

## Abbreviations

CNI: calcineurin inhibitors
CVD: cardiovascular disease
DAMPS: damage-associated molecular patterns
IRI: ischemia-reperfusion injury
KTR: kidney transplant recipient
MD: Mediterranean diet
NOX: NADPH oxidase
PTDM: post transplantation diabetes mellitus
RNS: reactive nitrogen species
ROS: reactive oxygen species
SOD: superoxide dismutase
TLR: Toll-like receptor.
